# Factors Predicting Variability in Total Small Bowel Length (TSBL) in People With Obesity Undergoing Metabolic Bariatric Surgery

**DOI:** 10.1007/s11695-026-08545-0

**Published:** 2026-02-26

**Authors:** Muffazal Lakdawala, Pooja Unadkat, Chetan Parmar, Nidhi Gandhi, Shreyans Rai, Jan Willem Greve

**Affiliations:** 1 Sir H.N. Reliance Foundation Hospital and Research Centre, Mumbai, India; 2 Department of Surgery, Whittington Health NHS Trust, London, UK; 3 University College London, London, UK; 4 Department of Academics and Research, Sir H.N. Reliance Foundation Hospital and Research Centre, Mumbai, India; 5 NUTRIM Research Institute of Nutrition and Translational Research in Metabolism, Maastricht University, Maastricht, Netherlands

**Keywords:** Total small bowel length (TSBL), Metabolic bariatric surgery (MBS), Bypass surgery

## Abstract

**Background:**

Variability in the length of the total small bowel has been published. There is no easy noninvasive method to measure the total small bowel length (TSBL), and intraoperative measures can be difficult and may lead to bowel injury. It is crucial to know the TSBL in patients undergoing bypass procedures for metabolic bariatric surgery (MBS). The present study aims to study the demographic, anthropometric and metabolic parameters that contribute to TSBL in patients undergoing MBS.

**Methods:**

Patients undergoing MBS at the single centre between Oct 2022-May 2025 were included. TSBL was measured from the duodenojejunal junction to the ileocaecal junction on unstretched bowel along the anti-mesenteric border by a single surgeon using lap instruments with markings. Data were statistically analyzed to find the correlation between TSBL and covariates. Weight loss and comorbidity outcomes were not measured for this study.

**Results:**

Total 246 consecutive patients were included in the study with 150 females. The mean age was 42.7 years, the mean height was 163.4 cm and the mean BMI was 45.7 kg/m^2^. The mean TSBL in the study participants was 797.7 cm. Height (*r* = 0.399) and presence of T2DM (p-value < 0.05) positively correlated, but age negatively correlated (*r* = -0.178) with TSBL. Linear regression analysis using age, sex, height, weight, dyslipidemia and diabetes to predict TSBL was carried and after adjusting for other significant factors, only age, height and diabetes were statistically significant to predict TSBL (p-value < 0.05).

**Conclusion:**

There is significant variability seen in TSBL in people with obesity. Height, and the presence of T2DM significantly impacted the length of TSBL positively, whereas advancing age negatively impacted TSBL. This highlights the need to measure TSBL in patients having a bypass as that would affect weight loss and comorbidity outcomes.

## Introduction

Obesity is a complex, chronic health condition characterized by the accumulation of excess fat that is associated with significant risk to overall health [1]. According to the World Health Organization (WHO), more than 1 billion people worldwide suffer from obesity [2]. A sedentary lifestyle and the easy availability of calorie-dense food further contribute to the rising global obesity rates [3]. Until genetic, environmental, behavioral, and socioeconomic factors are addressed, obesity will continue to plague humanity.

Metabolic and bariatric surgery (MBS) remains the best options for significant and sustained weight loss for patients with clinically severe obesity [4, 5]. The frequently performed MBS worldwide includes the sleeve gastrectomy (SG), Roux-en-Y gastric bypass (RYGB) and one anastomosis gastric bypass (OAGB) [6]. The important feature associated with bypass operations is the anatomical rearrangement that necessitates parts of the proximal small bowel to be bypassed, allowing for hormonal changes and the hypoabsorption of fats, proteins, multivitamins and micronutrients [7–11]. There is also evidence stating difference in weight loss depending on length of small bowel bypassed as a proportion of the total small bowel length (TSBL) [9, 10] and recurrent weight gain (RWG). This can be significant in proximal bypass procedures, compared to the distal bypass procedures wherein the length of common channel is known.

The small bowel plays a crucial role in the regulation of energy balance and metabolism via nutrient absorption, hormonal regulation, gut microbiota and bile acid metabolism, which together influence obesity [12, 13]. Understanding the interplay between the small bowel and mechanisms associated with obesity, coupled with pre-existing comorbid conditions helps tailor surgical approaches to individual patient needs and optimize weight loss and metabolic outcomes. It is thus crucial to know the TSBL in all patients undergoing bypass procedures.

There is considerable variability in the TSBL reported in literature, and there is no easy noninvasive method yet to measure TSBL preoperatively [14 –18]. Intraoperative measures are difficult to perform and may lead to bowel injury [19]. Bowel measurement is likely to have interobserver and interpersonal variability leading to inconsistencies in results [17–19]. The present study aims to determine the average TSBL in Indian patients with obesity undergoing bypass operations and analyse which of the selected demographic, anthropometric and metabolic parameters can influence the TSBL.

## Methods

The present study is a retrospective study. Approval was obtained from the institutional review board. It is routine practice in our unit to measure total small bowel length during MBS operation. No change in technique was made for this study. Hence no ethics approval was needed for this study. Data from all the eligible Indian patients with obesity undergoing MBS between October 2022 and May 2025 were extracted from the electronic database and analyzed to find the correlation between TSBL and other covariates. All consecutive patients undergoing gastric bypass surgery at the single centre were included. Patients undergoing Roux-en Y gastric bypass (RYGB), One Anastomosis gastric bypass (OAGB) and Sleeve plus procedures [Sleeve gastrectomy with duodeno-jejunal bypass (Sleeve-DJB) and Sleeve gastrectomy with proximal jejunal bypass (Sleeve-PJB)] were included in the study. Patients who had previous surgery on the small bowel, were undergoing revisional MBS or had inflammatory bowel disease were excluded from this study. The TSBL was measured using a pre-fixed marker at 5 cm on the laparoscopic hand instruments. The bowel was measured along the antimesenteric border in an unstretched fashion. All the measurements were done by the same surgeon, thereby decreasing variability. In our unit the bypassed small intestine is tailored based on the TSBL. For RYGB the alimentary limb is fixed, and the bilio-pancreatic limb (BPL) is one third and the common limb (CC) is two-third of the length. In OAGB, the BPL was one third of the TSBL.

### Demographic, Anthropometric, and Clinical Characteristics

Data were collected from participants for sex, age, height, weight, body mass index (BMI), and obesity related diseases such as hypertension, type 2 diabetes mellitus (T2DM), and dyslipidemia. Primary outcome was to measure the variability in the length of small bowel in patients. The secondary outcome was to see whether there was any correlation between the TSBL and other demographic variables.

### Statistical Tests

All statistical analyses were performed using STATA 17 (StataCorp. 2021. Stata Statistical Software: Release 17. College Station, TX: StataCorp LLC). After testing the normality of the data using the Shapiro-Wilk test, continuous variables were expressed as mean and standard deviation. The difference between the mean TSBL is compared to using the independent samples t-test. The correlation between the TSBL and the other selected demographic, anthropometric and metabolic parameters is compared using the Pearson Correlation coefficient. A multivariate analysis in the form of linear regression was done to predict the variability of TSBL based on selected covariates. A two-tailed p-value of < 0.05 was considered statistically significant for all analyses.

## Results

The study enrolled total of 246 participants.

Table 1 shows the demographic and anthropometric profile of the study participants. Of the 246 participants, 150 (60.98%) were females. The mean age of the group was 42.7 years; the mean height was 163.4 cm and the mean BMI was 45.73 kg/m^2^. More than 79% of the patients had a BMI of over 40 kg/m^2^.

**Table 1:** Demographic and anthropometric profile of the study participants

Factors	Frequency (Percentage)
Sex	
Females	150 (60.98%)
Males	96 (39.02%)
Age^1^ (years)	42.74 ± 12.61
Height^1^ (cms)	163.42 ± 10.10
Weight^1^ (kgs)	122.42 ± 23.80
BMI^1^ (Kg/m^2^)	45.73 ± 7.48
Obesity class	
Obesity I	18 (7.32%)
Obesity II	33 (13.41%)
Obesity III	132 (53.66%)
Obesity IV	50 (20.33%)
Obesity V	13 (5.28%)

*Note 1: Mean and standard deviation*

Overall, the mean TSBL in the study participants was 797.7 cm. The mean TSBL for females (775 cm), and for males (833.9 cm) was statistically significantly different. There was no significant difference in the TSBL based on comorbidities like hypertension and dyslipidemia. The mean TSBL was statistically significantly longer in patients with T2DM. This is shown in Table 2.

**Table 2:** Average TSBL based on sex and comorbidities

Covariates	*N*	TSBL	SD	*p*-value
Sex				0.004
Female	150	775.0	113.9	
Male	96	833.9	108.2	
Hypertension				0.557
No	145	795.2	114.3	
Yes	101	800.4	116.6	
T2DM				0.005
No	158	781.4	109.6	
Yes	88	826.9	119.4	
Dyslipidemia				0.057
No	166	787.6	113.6	
Yes	80	818.7	115.8	
Overall	246	797.7	115.1	

Table 3 shows the correlation between demographic factors and TSBL. Age was seen to be negatively correlated with TSBL (*r* = − 0.178), while height (*r* = 0.399) and weight (*r* = 0.168) were seen to be positively correlated with TSBL.

**Table 3:** Correlation between demographic and anthropometric factors and TSBL

Covariates	Correlation	*p*-value
Age	–0.178	0.005
Height	0.399	0.001
Weight	0.168	0.008
BMI	–0.102	0.109

Table 4 shows the unadjusted and adjusted variation in TSBL based on selected demographic factors, anthropometric factors and comorbidities. Unadjusted linear regression established that age, sex, height, weight, dyslipidemia and diabetes could statistically significantly predict TSBL (p-value < 0.05). However, after adjusting for other factors, sex, weight and dyslipidemia were not seen to be able to statistically significantly predict TSBL (p-value > 0.05). These factors accounted for approximately 20.9% of the explained variability in TSBL.

**Table 4:** Unadjusted and adjusted variation in TSBL based on selected demographic factors, anthropometric factors, and comorbidities

Total Bowel Length	Unadjusted Coefficient	Adjusted Coefficient
Age	–1.69 * (-2.86, -0.51)	− 1.41 * (-2.58, -0.25)
Sex	58.23 * (29.46, 86.99)	− 29.79 (-72.38, 12.79)
Height	4.56 * (3.24, 5.88)	5.36 * (3.30, 7.42)
Weight	0.81 * (-0.19, 0.27)	-0.10 (-0.77, 0.57)
BMI	–1.57 (-3.50, 0.36)	NA
T2DM	45.47 * (15.81, 75.14)	50.08 * (19.66, 80.50)
Hypertension	5.29 (-24.14, 34.72)	NA
Dyslipidemia	31.16 * (0.49, 61.82)	18.09 (-12.82, 49.00)
Constant	NA	-17.11
Note: *: p-value < 0.05		

## Discussion

This study of 246 consecutive patients had 60.9% females with mean age of 42.7 years and mean BMI of 45.73 kg/m^2^. The mean TSBL in the study participants was 797.7 cm. The mean TSBL was significantly longer in patients with T2DM. Age was seen to be negatively correlated with TSBL.

TSBL in humans has shown great variability [14, 16, 18] with a lot of factors affecting its length. Various methods have been employed to measure TSBL, ranging from preoperative CT scan with 3D volumetry, intraoperative measurements during surgery using either suture, tape or pre-marked instruments or cadaveric measurements [17, 19, 20, 21]. Bowel can be measured from the antimesenteric, mesenteric borders or in between. It can be measured either in the stretched or non-stretched way and the average difference reported between these 2 ways of measurement of bowel was 137 ± 19 cm (72 to 212 cm) [17].

Existing published literature has recorded the range of TSBL in to be between 253 and 1193 cm [15, 22, 23, 24, 25] and the average as 610 cm in females and 675 cm in males. The average TSBL reported by a study in Indian patients who underwent abdominal surgery was 777.1 cm [26]. In the present study, the mean TSBL was 797.7 cm (515 cm to 1125 cm), making it the study that has documented the longest average TSBL amongst other studies. Backman et al. reported a TSBL of 779 cm in patients with obesity, whereas the mean TSBL reported by other studies was shorter, namely, 463.5 cm reported by Hosseinpour et al., 506 cm by Teitelbaum et al., 579.5 cm by Dreike et al., 512 cm by Guzman et al., 562.5 cm by Nordgren et al., 608.5 cm by Honnou et al., 615 cm by Underhill et al., 625 cm by Bryant et al., and 698.5 cm by Treves et al. [14, 22, 23, 27–33]. The shortest TSBL reported in the present study was 515 cm, which is nearly double the shortest length reported in literature of 253 cm in patients with obesity and 201 cm in non-obese individuals, whereas the longest TSBL of 1125 cm was marginally shorter than that reported in literature of 1193 cm [17, 22, 23]. Some possible reasons for the variability may be the smaller sample size of the studies or variability in how the TSBL was measured.

### Sex

Studies have reported that 3% of males and 2% of females have TSBL less than 400 cm, and 15% of males and 5% of females have TSBL more than 800 cm [17]. In the present study, it was observed that the mean TSBL in men was 833.9 cm, and it was longer than 775 cm found in women. However, on multivariate linear regression, after adjusting for other variables, there was no statistically significant difference seen in the mean TSBL between females and males. Other studies showed similar results to this study. Underhill et al. showed the mean TSBL for males as 638 cm as compared to 592 cm for females, Dreike et al. males 633 cm, females 526 cm, Nordgren et al. males 591 cm, females 534 cm, Hounnou et al. males 644 cm, females 573 cm, Byrant et al. males 663 cm, females 587 cm [14, 29, 30, 31, 32]. Only 2 studies (Hosseinpour males 459 cm, females 468 cm and Treves et al. males 686 cm, females 711 cm) showed females to have longer TSBL than males [27, 33]. The variation in the TSBL in men and women should be factored in when deciding the length of the BPL to be bypassed.

### Age

**Fig. 1:**
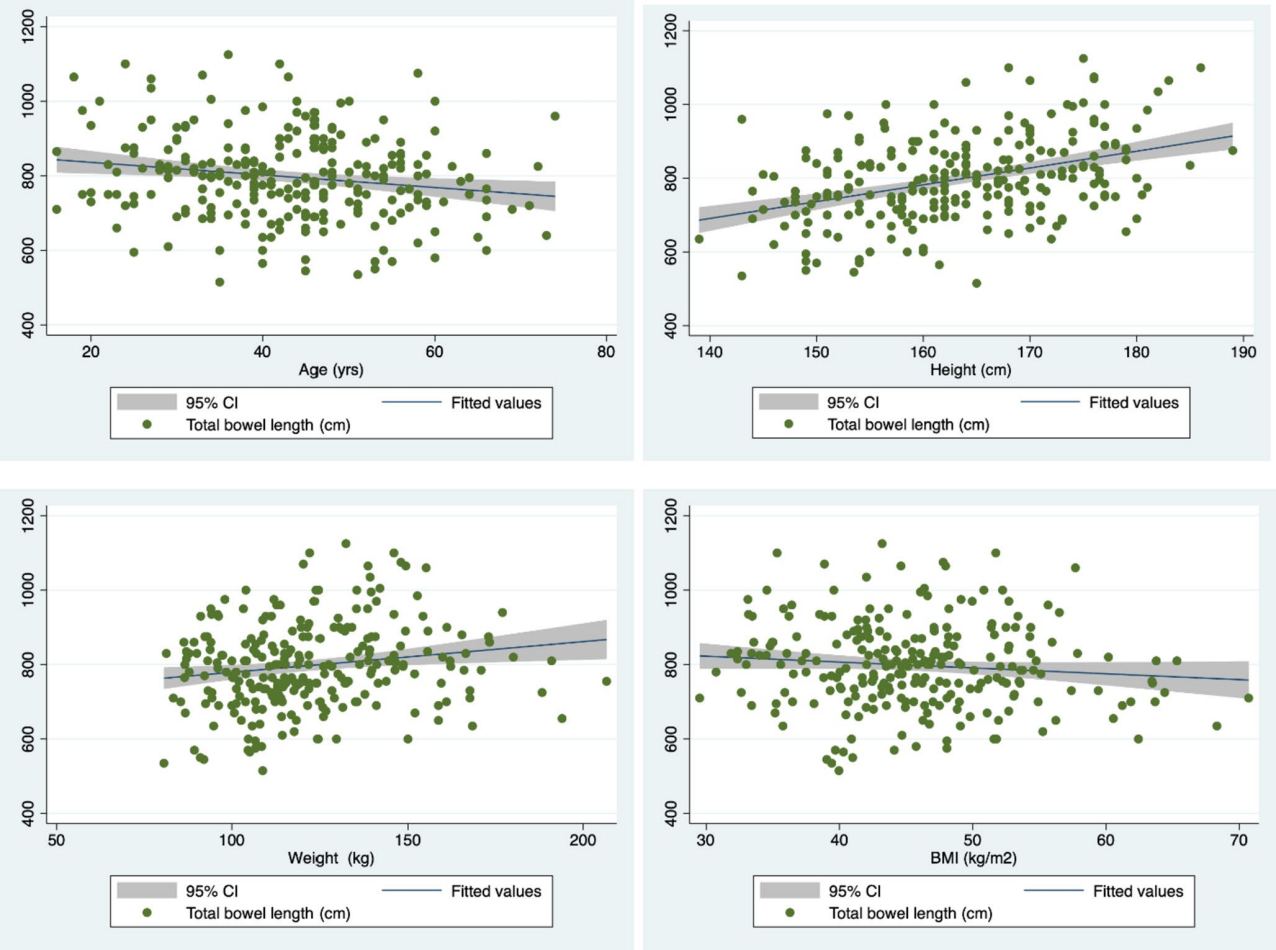
Scatterplot of TSBL with age, height, weight, and BMI

The relationship between age and TSBL has not been constant across literature [17]. In the present study age was seen to be negatively correlated with TSBL [Table 3; Fig. 1]. TSBL was found to be shorter in older patients. Similarly, a negative correlation was seen between age and TSBL in studies by Hounnou and colleagues conducted on cadavers [31]. Whereas Teitelbaum et al. reported that TSBL had a negative correlation with age only for women whereas a positive correlation was seen for men [28]. In studies by Bekheit et al., Hosseinpur et al., Nordgren et al. and Treves et al., no correlation was found between age and TSBL [14, 25, 27, 30, 33]. Based on this study it is worth remembering that the TSBL is shorter in older patients. Hence it is safer to avoid long limb length being bypassed in this age group patients to prevent any malnutritional issues as it can prove fatal.

### Height

Studies across literature have suggested a positive correlation between height and TSBL [14, 30, 33]. In the present study height was seen to be positively correlated with TSBL. Height was a statistically significant determinant of TSBL even on multivariate analysis after adjusting for other variables. Tietelbaum in their study found a correlation between height and TSBL only in their female subgroup [28]. Treves, Drieke, Hosseinpur and Honnou et al. however found no correlation between height and TSBL ^[,27,29,31,33]^ in both males and females. This helps us conclude that in taller patients the surgeons have option to bypass longer limb length if they wish to have higher weight loss. However, the common channel length should always be measured when doing so to avoid risk of nutritional issues.

### Weight and BMI

In the present study, a positive correlation was observed between weight and TSBL. However, on multivariate linear regression after adjusting for other variables it was not a statistically significant determinant of TSBL. Nordgren et al. and Honnou et al. were the only other studies that showed a positive correlation between weight and TSBL [30, 31]. Backman et al. and Guzman et al. measured TSBL in patients with obesity and in the non-obese individuals. Backman and colleagues showed an average of 779 cm in patients with obesity and 657 cm in non-obese individuals, whereas Guzman and colleagues reported 562 cm in males and 502 in females who were obese and 530 cm in males and 507 cm in females in the non-obese group [22, 23]. Thus, showing that individuals with obesity probably have longer TSBL. No statistically significant correlation was found between BMI and TSBL in this study, which is consistent with what has been reported by other studies ^[9,30,313]^. These findings help justify the arguments of surgeons who are proponents of tailoring the limb length according to the BMI of the patients [30].

### Metabolic Syndrome; T2DM, Hypertension and Dyslipidemia

Our study found that patients with diabetes have statistically significantly longer TSBL. The only other study to have observed a significant but weak positive correlation between TSBL and T2DM was conducted in Indian patients undergoing an open or laparoscopic abdominal procedure. The study highlighted that patients with an HbA1c ≥ 6.5% and random blood sugar ≥ 200 mg/dL had significantly longer TSBL [29]. While studies can attest to the diabetes associated histomorphological and mechanical changes in the intestine, the relationship between TSBL and diabetes has never been studied and needs to be explored further [34]. Some surgeons bypass longer limb length in patients with T2DM and our study supports their argument. Other metabolic diseases such as hypertension, however, had no significant impact on the mean TSBL. Dyslipidemia, on multivariate linear regression after adjusting for other variables. was not a statistically significant determinant of TSBL.

This finding gives food for thought to studies that have theorized that outcomes of MBS may vary depending on the TSBL of the patient [18, 35]. While this makes for an interesting hypothesis, the relationship between TSBL and MBS outcomes needs to be explored further in prospective large studies by altering the length of TSBL bypassed as a % of the TSBL for any proximal bypass operation such as the RYGB, OAGB, Sleeve Duodeno-Jejunal Bypass (LSG + DJB) or the Sleeve Gastro-Jejunal Bypass (SASJ). There is evidence that longer the biliopancreatic limb (BPL) the better the weight loss, and remission of comorbidities [30,36, 37]. However, it also increases the risk of protein, multivitamin and micronutrient deficiencies along with side effects like SIBO and steatorrhea [38, 39, 40, 41].

The variability in TSBL thus makes the ideal length of small intestine that must be bypassed a debatable topic. Every patient’s response to MBS is subjective, and dependent on a lot of factors other than TSBL alone [42–48], one example being different absorption and hormonal function of jejunum and ileum.

Hence it a good option to measure TSBL in all patients undergoing a bypass surgery given its variability to determine the appropriate length of proximal jejunum to be bypassed in relation to the TSBL. Observations from this study helps us hypothesize that when performing a standard bypass surgery on taller and diabetic patients, it would be advisable to have a relatively longer BPL in order to achieve optimal weight loss and remission of comorbidities. Similarly, in the case of older and shorter patients, since the TSBL would be shorter, it would be safe to have a relatively shorter BPL, to prevent protein energy malnutrition, steatorrhea, etc. With the popularity of GLP-1 medications and bipartition operations there is increased interest in debate whether a ‘’gastro-jejunal’’ or a ‘’gastro-ileal’’ anastomosis would give better and safer outcomes [49, 50]. Some surgeons perform ‘’fixed’’ BPL. Whereas others vary limb length according to the BMI but might not measure the whole small bowel. We inadvertently keep hearing fatal reports on malnutrition and contrary to this suboptimal outcome or recurrent weight gain in some patients. Hence, we wanted to highlight the variability of limb length again and remind the readers that it should be considered when making decision to bypass the limb length.

It is our endeavor to expand this study to statistically significant numbers to arrive at a mathematical formula that can measure TSBL preoperatively in patients with a high percentage of sensitivity and specificity. There are certain limitations of this study. One limitation of this study is its manual measurements of TSBL which can have variations. However, that variability is minimized as it’s been done at a single center by the same surgeon using the same instruments and technique of measurement. The other limitation is that while we have shown marked differences in TSBL, we do not have any data on how to use these measurements to tailor the limb lengths per patient or how much of Jejunum or ileum needs to be bypassed as the physiological function of both varies. We have also not discussed the physiological role of Jejunum or ileum. This is beyond the remit of this study as we have not discussed weight loss or comorbidity resolution outcomes in this study. However, we believe that this study would act as a basis on which such large robust study can be designed in the future.

## Conclusion

This study concludes that height, and presence of diabetes positively impacted TSBL, whereas advancing age negatively influenced TSBL, in Indian people with obesity. There remains a variability of 20.9% in TSBL despite accounting for various variables. This knowledge would be able to answer at least a part of the puzzle as to why different people get different results with the concept of a fixed BPL when performing bypass operations.

## Data Availability

No datasets were generated or analysed during the current study.
